# The Roles of Organic Acids in C_4_ Photosynthesis

**DOI:** 10.3389/fpls.2016.00647

**Published:** 2016-05-17

**Authors:** Martha Ludwig

**Affiliations:** School of Chemistry and Biochemistry, The University of Western Australia, CrawleyWA, Australia

**Keywords:** C_4_ photosynthesis, C_4_ acid, malate, aspartate, NAD-malic enzyme, NADP-malic enzyme, oxaloacetate, phospho*enol*pyruvate carboxykinase

## Abstract

Organic acids are involved in numerous metabolic pathways in all plants. The finding that some plants, known as C_4_ plants, have four-carbon dicarboxylic acids as the first product of carbon fixation showed these organic acids play essential roles as photosynthetic intermediates. Oxaloacetate (OAA), malate, and aspartate (Asp) are substrates for the C_4_ acid cycle that underpins the CO_2_ concentrating mechanism of C_4_ photosynthesis. In this cycle, OAA is the immediate, short-lived, product of the initial CO_2_ fixation step in C_4_ leaf mesophyll cells. The malate and Asp, resulting from the rapid conversion of OAA, are the organic acids delivered to the sites of carbon reduction in the bundle-sheath cells of the leaf, where they are decarboxylated, with the released CO_2_ used to make carbohydrates. The three-carbon organic acids resulting from the decarboxylation reactions are returned to the mesophyll cells where they are used to regenerate the CO_2_ acceptor pool. NADP-malic enzyme-type, NAD-malic enzyme-type, and phospho*enol*pyruvate carboxykinase-type C_4_ plants were identified, based on the most abundant decarboxylating enzyme in the leaf tissue. The genes encoding these C_4_ pathway-associated decarboxylases were co-opted from ancestral C_3_ plant genes during the evolution of C_4_ photosynthesis. Malate was recognized as the major organic acid transferred in NADP-malic enzyme-type C_4_ species, while Asp fills this role in NAD-malic enzyme-type and phospho*enol*pyruvate carboxykinase-type plants. However, accumulating evidence indicates that many C_4_ plants use a combination of organic acids and decarboxylases during CO_2_ fixation, and the C_4_-type categories are not rigid. The ability to transfer multiple organic acid species and utilize different decarboxylases has been suggested to give C_4_ plants advantages in changing and stressful environments, as well as during development, by facilitating the balance of energy between the two cell types involved in the C_4_ pathway of CO_2_ assimilation. The results of recent empirical and modeling studies support this suggestion and indicate that a combination of transferred organic acids and decarboxylases is beneficial to C_4_ plants in different light environments.

## Introduction

Organic acids are of fundamental importance in all plant species. They are involved in many and diverse metabolic pathways, including energy production, carbon storage, stomatal conductance, the biosynthesis of amino acids, plant–microbe interactions, and mechanisms allowing plants to deal with excess cations, changing osmotic conditions, and soils low in nutrients as well as those with high metal content (reviewed in López-Bucio et al., 2000). In addition to these varied roles, organic acids play a major part in the C_4_ photosynthetic pathway as the intermediates connecting CO_2_ uptake and fixation, and this is the focus of this review.

Work on sugarcane published in the 1960s (Burr, 1962; Kortschak et al., 1965; Hatch and Slack, 1966; Hatch et al., 1967) showed that this plant performed a different type of CO_2_ fixation biochemistry compared to the photosynthetic carbon reduction (PCR) cycle determined in the Calvin laboratory (Bassham and Calvin, 1957). ^14^CO_2_ labeling studies indicated the first major products of CO_2_ assimilation in sugarcane were the 4-carbon (C) organic acids malate and aspartate (Asp) rather than 3-phosphoglycerate (3-PGA) or other intermediates of the PCR cycle (Burr, 1962; Kortschak et al., 1965; Hatch and Slack, 1966; Hatch et al., 1967). Hatch and Slack (1966) described a model of the pathway, which encompassed and extended the earlier work. The pathway is now known as the C_4_ pathway because of the initial fixation products, and plants using the pathway are known as C_4_ plants. This seminal work (Hatch and Slack, 1966) also resolved that the fourth C of oxaloacetate (OAA) was labeled along with those of malate and Asp. However, OAA was short-lived, and immediately converted to malate or Asp. The label in these dicarboxylic acids was transferred to the first C of 3-PGA, which then was used for carbohydrate synthesis via the PCR cycle. Their model also included a 3-C organic acid as the acceptor molecule for atmospheric CO_2_ (Hatch and Slack, 1966).

**FIGURE 1:**
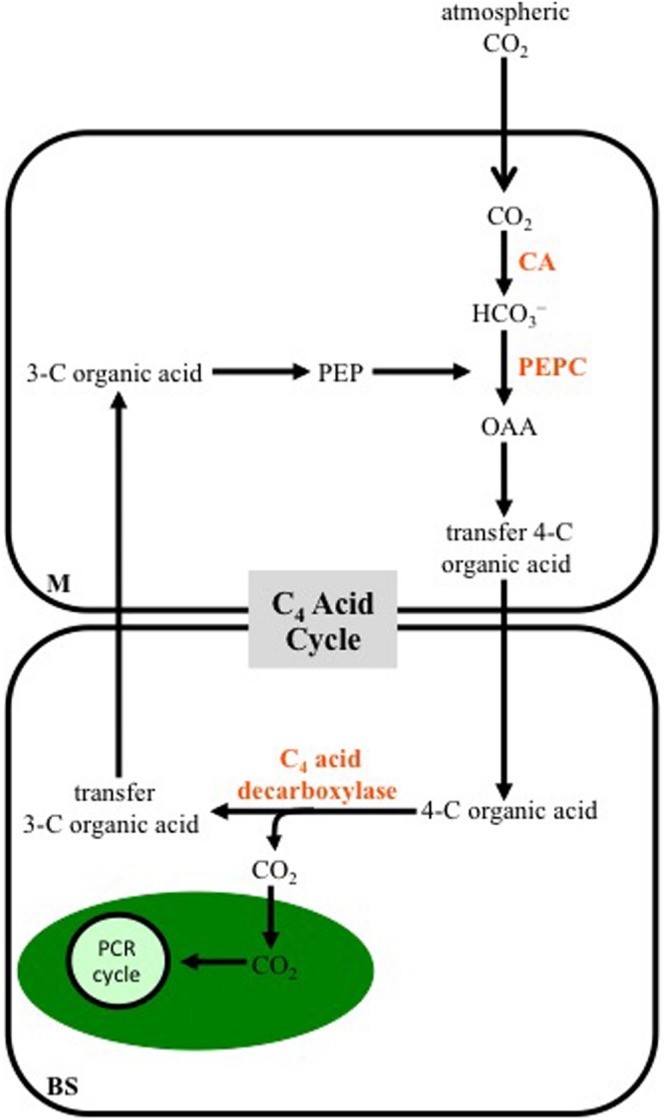
**A diagram outlining a generalized C_4_ photosynthetic pathway.** Atmospheric CO_2_ enters a mesophyll (M) cell of a leaf and is converted to bicarbonate (HCO_3_^-^) by carbonic anhydrase (CA). The bicarbonate is then used to carboxylate phospho*enol*pyruvate (PEP) by PEP carboxylase (PEPC), producing oxaloacetate (OAA), which is immediately converted into another four-carbon (C) organic acid that is transferred to bundle-sheath (BS) cells. Decarboxylation of the 4-C acid occurs in BS cells, releasing CO_2_, which is fixed into carbohydrates by the photosynthetic carbon reduction (PCR) cycle in the chloroplast (green oval). The 3-C organic acid released at the decarboxylation step, is transferred to M cells, where it contributes to the CO_2_ acceptor pool. Note the 3-C and 4-C organic acids formed, and the intracellular location of the decarboxylation reactions vary, depending on the C_4_ subtype (see **Figures 2–4**).

A generalized scheme of the reactions making up the C_4_ photosynthetic pathway is shown in **Figure 1**. Unlike C_3_ plants, most C_4_ species use two cell types in CO_2_ assimilation: the mesophyll (M) and bundle-sheath (BS) cells. The BS cells surround the vascular tissue, and are in turn surrounded by M cells, giving the characteristic wreath-like or Kranz anatomy that was first describe in the late 19th century (Haberlandt, 1896). In C_4_ leaves, no M cell is more than two cells away from a BS cell, which facilitates rapid metabolite exchange between the two cell types. Atmospheric CO_2_ entering a C_4_ leaf is hydrated to bicarbonate in the M cytosol by carbonic anhydrase (CA). The primary carboxylase of C_4_ plants, phospho*enol*pyruvate carboxylase (PEPC), uses the bicarbonate to fix a CO_2_ group to the 3-C compound phospho*enol*pyruvate (PEP), producing OAA, which is rapidly converted to malate and/or Asp. These dicarboxylic acids move into the neighboring BS where they are decarboxylated, with the released CO_2_ being used for carbohydrate production by the PCR cycle, located in BS cell chloroplasts. The 3-C organic acids released in the decarboxylation reactions return to a neighboring M cell, where they can be used as the CO_2_ acceptor by PEPC (**Figure 1**).

An important outcome of the C_4_ acid cycle is the concentration of CO_2_ around ribulose-1,5-bisphosphate carboxylase/oxygenase (Rubisco) in the BS to levels at least 10-times higher than those of the surrounding atmosphere (Jenkins et al., 1989). This results in C_4_ plants requiring less Rubisco for carbohydrate production than C_3_ species, which translates into increased nitrogen-use efficiency (Ghannoum et al., 2011). In contrast to Rubisco, which can fix oxygen in addition to CO_2_, leading to photorespiration and the loss of fixed CO_2_ and consumption of ATP, PEPC has only carboxylase activity. Consequently C_4_ plants have little photorespiratory activity, and through a combination of PEPC kinetics and leaf anatomy, also show increased water-use efficiency compared to C_3_ plants (Ghannoum et al., 2011).

As more C_4_ plants were examined, it became clear that while the first two steps in the pathway, those catalyzed by CA and PEPC, are the same in all species (Hatch, 1987; Kanai and Edwards, 1999), differences exist in the 4-C organic acid transferred to the BS; the decarboxylating enzymes and their activities and intracellular locations; the 3-C organic acid returned to the M cells; and regeneration of the CO_2_ acceptor. As a result, three subtypes of C_4_ photosynthesis were recognized (Gutierrez et al., 1974; Hatch et al., 1975; Hatch, 1987; Kanai and Edwards, 1999), based on the decarboxylating enzyme with the greatest activity in the leaf tissue, and C_4_ species were categorized into one of these subtypes: NADP-malic enzyme (NADP-ME), NAD-malic enzyme (NAD-ME), or PEP carboxykinase (PCK).

This review will summarize the three C_4_ subtypes, focusing on the organic acids utilized in the course of CO_2_ fixation, and the enzymes responsible for their metabolism. The roles of the 4-C organic acid decarboxylases in C_3_ plants will be presented along with the current understanding of their co-option into C_4_ biochemistry. Consideration will then be given to evidence suggesting that C_4_ plants are more flexible with respect to the types of organic acids and decarboxylases used than previously thought, and the apparent advantages this plasticity gives the plants in fluctuating environments will be discussed.

## C_4_ Photosynthetic Subtypes

### NADP-ME-Subtype

**FIGURE 2:**
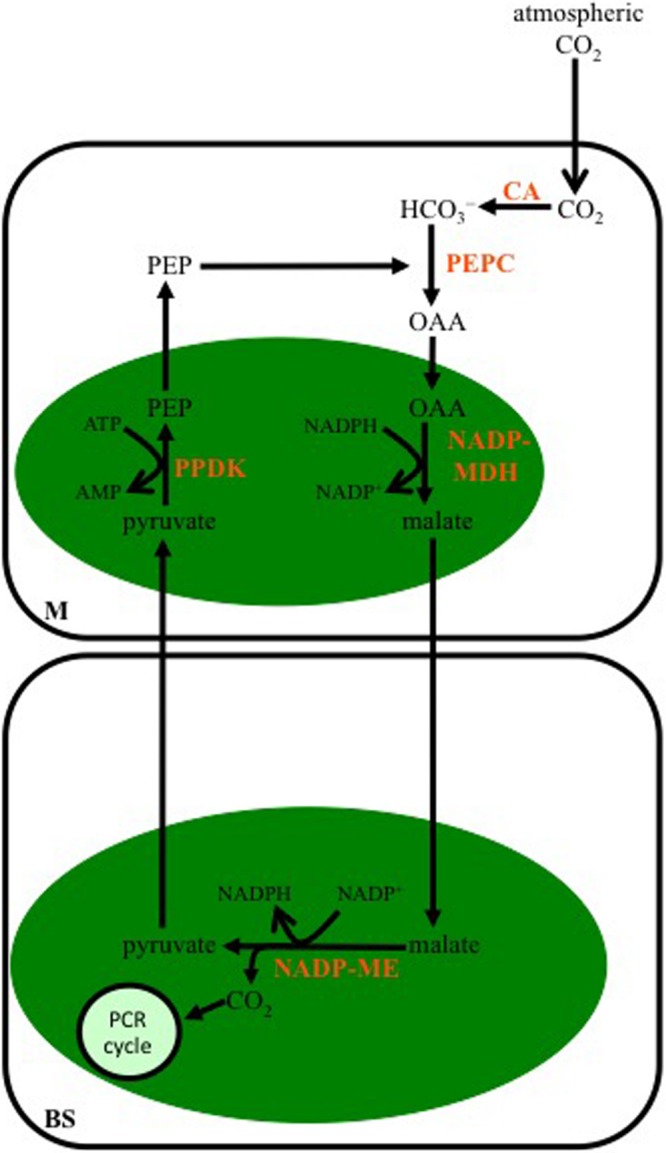
**Schematic of the C_4_ NADP-malic enzyme-subtype pathway.** In mesophyll (M) cells of this C_4_ subtype, the bicarbonate (HCO_3_^-^) produced from atmospheric CO_2_ by CA is used to carboxylate phospho*enol*pyruvate carboxylase (PEP) by PEPC. The OAA formed is reduced to malate in M chloroplasts (green oval), by NADP-malate dehydrogenase (NADP-MDH), and then diffuses into BS cells, where it is decarboxylated in the chloroplast by NADP-malic enzyme (NADP-ME). The CO_2_ released is used to make carbohydrates by the PCR cycle, while the pyruvate diffuses to the M where it is converted to PEP by pyruvate Pi dikinase (PPDK).

Malate is the major 4-C organic acid transferred to the BS in C_4_ species designated as belonging to the NADP-ME subtype (**Figure 2**). The OAA synthesized from PEPC activity in the cytoplasm of M cells is transferred to the chloroplast and reduced to malate by NADP-malate dehydrogenase (NADP-MDH). The malate is then transported out of the M chloroplasts, diffuses into the BS, and in the BS chloroplasts is decarboxylated by NADP-ME in a reaction producing NADPH, CO_2_, and pyruvate. The released CO_2_ is fixed by Rubisco in the BS chloroplasts, while the pyruvate is transported out of the BS chloroplasts, and into the chloroplasts of M cells, where it is used to regenerate PEP by pyruvate Pi dikinase (PPDK).

The NADP-ME subtype is the most prevalent C_4_ subtype, and is found in both monocotyledonous and dicotyledonous species (Gutierrez et al., 1974; Sage et al., 2011). Agronomically important monocots such as maize, sorghum, and sugarcane are categorized as NADP-ME species. C_4_ species of *Flaveria*, a dicotyledonous genus that has been used to examine the evolution of C_4_ photosynthesis for more than 25 years, also show high levels of malate production during CO_2_ fixation, as well as high activity of NADP-ME in leaf tissue.

### NAD-ME-Subtype

C_4_ plants using NAD-ME as their primary decarboxylase include *Atriplex*, and C_4_ species of *Cleome* and *Amaranthus* (Gutierrez et al., 1974; Sage et al., 2011). Some C_4_ species of *Panicum* also have high NAD-ME activity in BS mitochondria.

**FIGURE 3:**
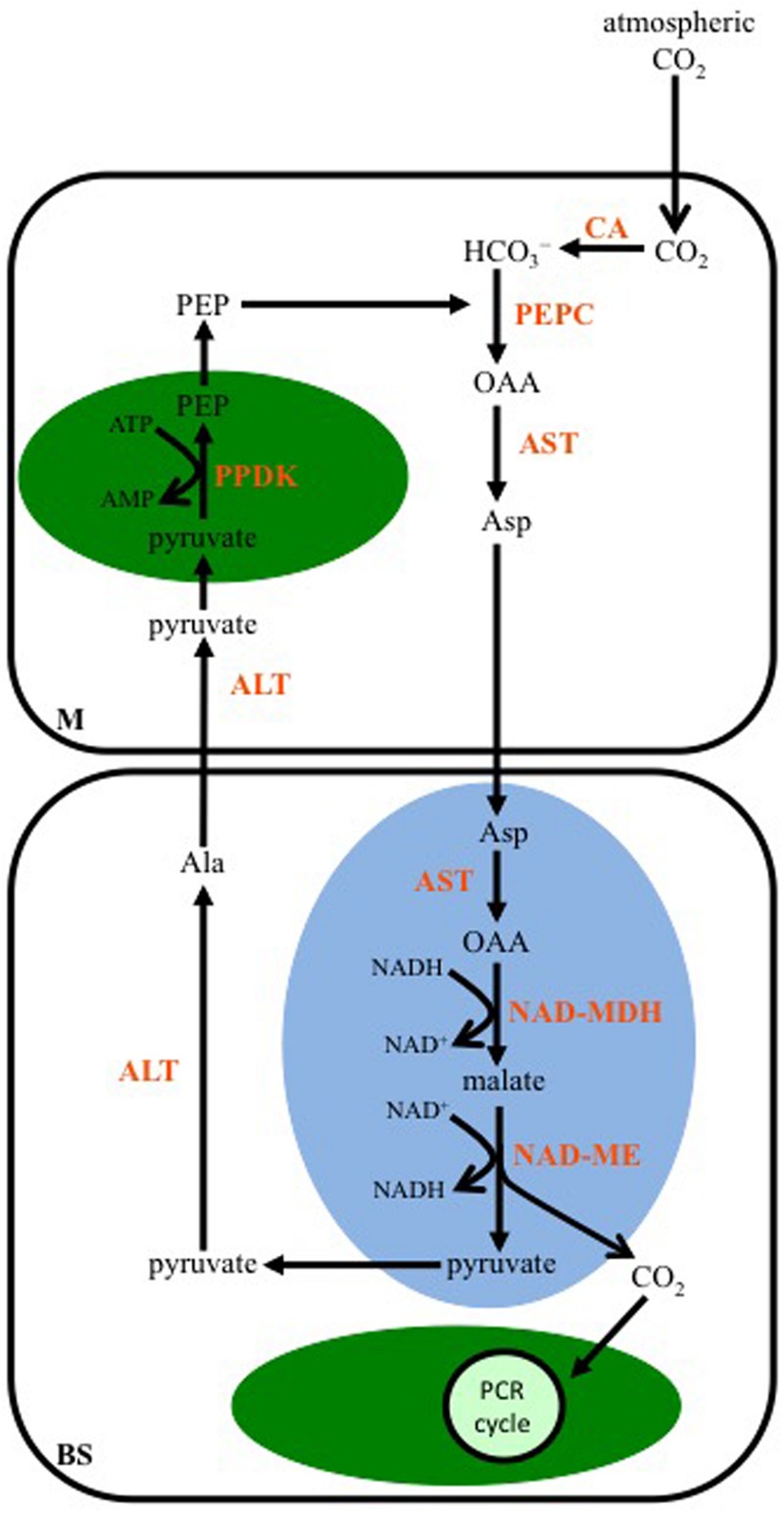
**A diagram showing the steps in the C_4_ NAD-malic enzyme-subtype pathway.** In mesophyll (M) cells, PEP is carboxylated by PEPC using the CO_2_ group from bicarbonate (HCO_3_^-^), which was produced from the hydration of atmospheric CO_2_ by CA. The resulting OAA is transaminated to Asp by Asp aminotransferase (AST). In the mitochondria (blue oval) of BS cells, OAA is generated from Asp by a mitochondrial AST, and then reduced to malate by NAD-malate dehydrogenase (NAD-MDH). Malate is decarboxylated by NAD-malic enzyme (NAD-ME), and the released CO_2_ diffuses into the BS chloroplast (green oval), where it is fixed by the PCR cycle. The pyruvate resulting from the decarboxylation reaction is converted to alanine (Ala) by Ala aminotransferase (ALT) in the BS cytosol. The Ala is transaminated back to pyruvate by ALT activity in the M cytosol. PPDK activity in M chloroplasts converts pyruvate to PEP.

Aspartate is the first stable organic acid of this C_4_ subtype, and results from the transamination of OAA by an Asp aminotransferase (AST) located in the cytosol of M cells (**Figure 3**). The Asp enters the mitochondria of BS cells where it is converted back to OAA by a mitochondrial isoform of AST, and through the activity of a mitochondrial NAD-malate dehydrogenase (NAD-MDH), the OAA is reduced to malate. NAD-ME is also active in the BS mitochondria, and catalyzes the release of CO_2_ from malate, along with the reduction of NAD^+^. The CO_2_ diffuses into the chloroplasts of the BS, and is fixed by Rubisco. The pyruvate, resulting from the decarboxylation reaction, is transported out of the mitochondria, and transaminated to Ala by a cytosolic Ala aminotransferase (ALT). The Ala diffuses into a neighboring M cell, and is converted back to pyruvate in the reverse reaction catalyzed by an ALT active in the M cell cytosol. The pyruvate is then used by PPDK to regenerate PEP in M cell chloroplasts.

### PCK-Subtype

**FIGURE 4:**
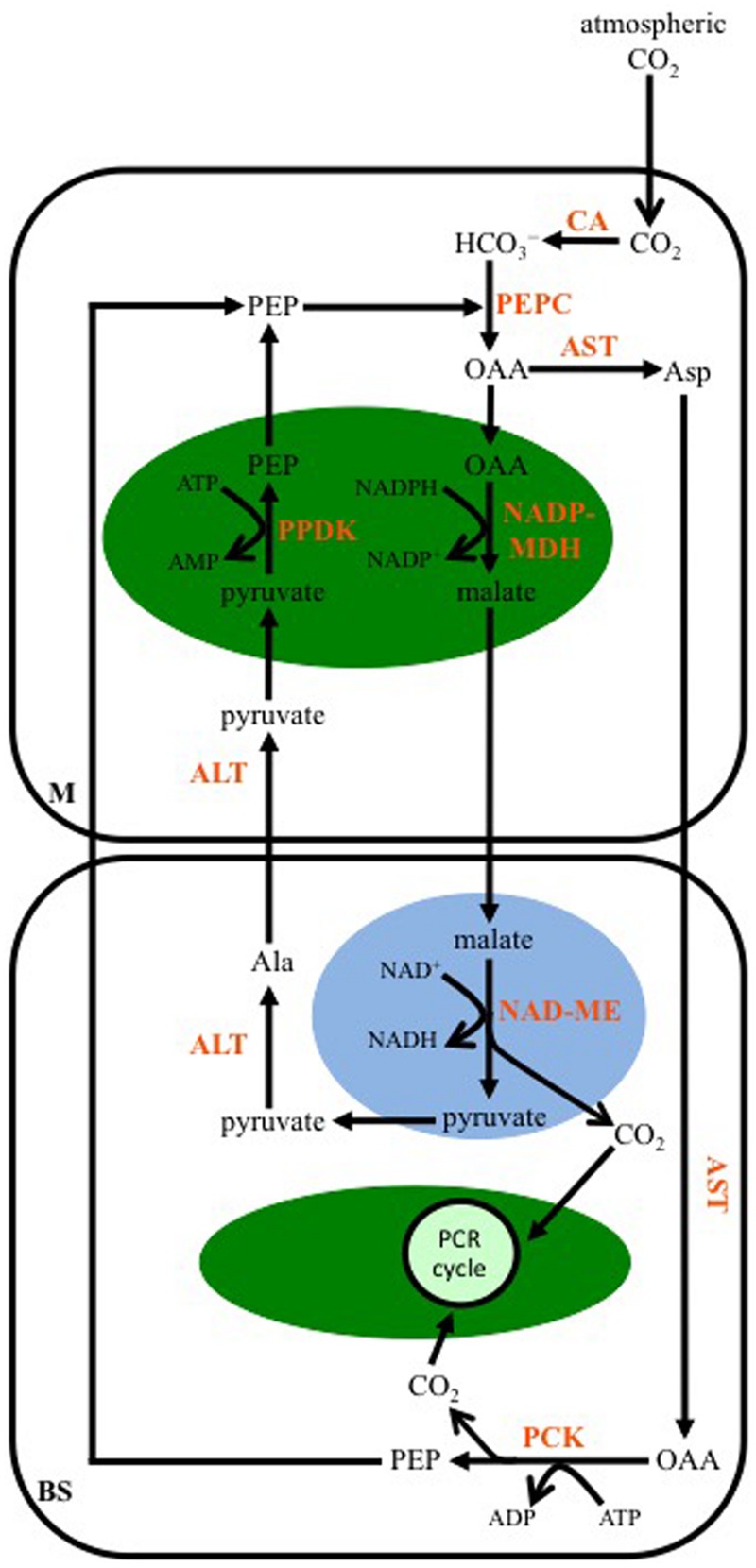
**A representation of the C_4_ phospho*enol*pyruvate carboxykinase-subtype pathway.** Atmospheric CO_2_ is converted to bicarbonate (HCO_3_^-^) by CA in the mesophyll (M) cytosol. PEP is carboxylated, producing OAA, by PEPC. The OAA is either transaminated to Asp by AST in the cytosol, or is reduced to malate in the chloroplast (green oval) by NADP-MDH. The Asp diffuses into the BS cell cytosol, where it is transaminated back to OAA, which is then decarboxylated by PEP carboxykinase (PCK). The resulting PEP diffuses into the M, while the CO_2_ is used to make carbohydrates by the PCR cycle. The malate formed in M chloroplasts is transferred to BS mitochondria (blue oval), where it is decarboxylated by NAD-ME, with the released CO_2_ diffusing into BS chloroplasts for fixation, and the pyruvate being transaminated to Ala in the BS cytosol by ALT. The Ala is transferred to the M, where another ALT converts it back to pyruvate, which is then converted to PEP in the chloroplast by PPDK.

As for the NAD-ME-subtype, multiple transamination reactions characterize the C_4_-PCK-subtype pathway. In plants using this enzyme as their primary decarboxylase, both malate and Asp are formed from OAA (**Figure 4**). A cytosolic AST in M cells produces Asp, while OAA is also transported into M cell chloroplasts and converted to malate by NADP-MDH. The Asp diffuses into the BS, and there a cytosolic AST converts it back to OAA, which is then decarboxylated by PCK in a reaction requiring ATP. The released CO_2_ enters the PCR cycle in the BS chloroplasts. The PEP generated from PCK activity diffuses back into the M to be used by PEPC. The malate formed in M cell chloroplasts is transported out of these organelles, and into the mitochondria of the BS. As for the NAD-ME-subtype, a mitochondrial NAD-ME isoform decarboxylates the malate, the released CO_2_ enters BS chloroplasts and the PCR cycle. The pyruvate formed is ultimately used to regenerate PEP in the M cell chloroplasts, following the same transamination reactions as those of the NAD-ME-subtype pathway. The NADH produced from NAD-ME activity is used in mitochondrial respiration to make ATP, supporting PCK activity in the BS cytosol.

PCK-type grasses include *Urochloa panicoides, Chloris gayana*, and some C_4_ species of *Panicum* (Gutierrez et al., 1974; Sage et al., 2011). It was thought PCK did not play a role in CO_2_ assimilation in C_4_ dicots; however, significant PCK activity has been reported in several C_4_ dicot lineages, including members of the Sesuvioideae and *Cleome* (Meister et al., 1996; Muhaidat et al., 2007; Sommer et al., 2012; Muhaidat and McKown, 2013). In contrast, a more recent study examining C_4_ species in the Cleomaceae, Aizoaceae, and Chenopodiaceae detected only low levels of PCK in these dicot groups (Koteyeva et al., 2015).

## Evolution of C_4_ Acid Decarboxylases

All the C_4_ cycle enzymes involved in the production and utilization of malate, OAA, Asp, Ala, PEP, and pyruvate have counterparts in C_3_ species, as these organic acids are involved in a myriad of roles in all plants, as noted above (López-Bucio et al., 2000). Many of the genes encoding the C_4_ proteins appear to have resulted from duplication events, which allowed ancestral function to be maintained, while also permitting neofunctionalization of the other copy, leading to the C_4_-specific roles and expression patterns (Ludwig, 2013). Interestingly, a number of the genes encoding the enzymes involved in the metabolism of the organic acids appear to have been co-opted from the same gene lineage in numerous independent C_4_ lineages (Christin et al., 2013, 2015). Changes in regulatory mechanisms of the ancestral genes, which may involve sequences in the promoter, untranslated and/or coding regions, and *trans*-acting factors led to the distinctive C_4_ expression levels and cell-specific patterns, while modifications to the coding regions were responsible for differences in the kinetic properties seen between the C_3_ and C_4_ enzymes (Williams et al., 2012; Ludwig, 2013). The evolution of the decarboxylases catalyzing the release of CO_2_ from either malate or OAA in the three C_4_ subtypes has been studied to varying levels, and our current knowledge is summarized below.

### NADP-Malic Enzyme

Cytosolic and chloroplastic forms of NADP-ME exist in both C_3_ and C_4_ plants. The cytosolic proteins play roles in defense, development, and stress responses by coordinating the levels of malate, pyruvate, and reducing power needed by a plant during these processes (**Table 1**) (Drincovich et al., 2011; Maier et al., 2011; Badia et al., 2015). These cytosolic enzymes are thought to represent the ancestral form of the protein. It has been suggested for maize that the gene encoding a cytosolic NADP-ME was duplicated, with one of the resulting copies acquiring a sequence encoding a chloroplast transit peptide (Tausta et al., 2002). A duplication of this gene led to the C_4_ isoform with its ability to decarboxylate malate in BS chloroplasts, and its characteristic properties of tetramerization and regulation, including inhibition by high malate concentrations at pH 7 and redox modulation (Detarsio et al., 2007; Alvarez et al., 2012; Saigo et al., 2013).

**Table 1:** Characteristics of four-carbon organic acid decarboxylating enzymes in the leaves of C_4_ plants and the orthologous gene products from C_3_ species.

Enzyme	Location / function in C_3_ leaf cells	Location / function of C_4_ isoforms in bundle-sheath cells	Identified changes in the genes encoding C_4_-associated isoforms / species (reference)	Identified differences in C_4_ isoforms / species (reference)
NADP-ME	Cytosol/defense, development, stress response Chloroplasts / lipid and protein biosynthesis, nitrogen assimilation, defense responses	Chloroplasts / major decarboxylase in NADP-ME-type C_4_ species	High BS-specific expression controlled by sequences in the upstream region, 5′-coding and 3′-UTR / *Flaveria bidentis* (Marshall et al., 1997; Ali and Taylor, 2001) High BS-specific expression controlled by sequences in the upstream region */ Flaveria trinervia* (Lai et al., 2002)	Malate inhibition / *Zea mays*; *Sorghum bicolor* (Detarsio et al., 2007; Saigo et al., 2013) Redox modulation / *Zea mays*; *Sorghum bicolor* (Alvarez et al., 2012; Saigo et al., 2013) Tetramerization / *Zea mays*; *Sorghum bicolor* (Detarsio et al., 2007; Saigo et al., 2013)
NAD-ME	Mitochondria / respiration of malate	Mitochondria / major decarboxylase in NAD-ME-type C_4_ species	Sequences at 5′-end of coding region responsible for BS-specific expression */ Gynandropsis gynandra* (Brown et al., 2011)	None identified
PCK	Cytosol / gluconeogenesis, seedling development, stomatal control, replenishment of TCA intermediates, nitrogen metabolism, pH regulation	Cytosol / major decarboxylase in PCK-type C_4_ species	None identified	None identified

*BS, bundle-sheath; n/a, not applicable; NAD-ME, NAD-malic enzyme; NADP-ME, NADP-malic enzyme; PCK, phospho*enol*pyruvate carboxykinase; UTR, untranslated region.*

A similar duplication of the gene encoding a chloroplastic, non-C_4_-associated enzyme has been proposed for the evolution of the C_4_ NADP-ME in *Flaveria* (Marshall et al., 1996). Potentially interacting regulatory elements found in the 5′- and 3′-ends of the *F. bidentis* gene encoding the C_4_-associated NADP-ME control the level of gene activity, while other sequences in the 5′-end were found to determine BS specificity (**Table 1**) (Marshall et al., 1997; Ali and Taylor, 2001). In the closely related C_4_ species *F. trinervia*, the high levels of BS-specific expression of the C_4_-associated NADP-ME are apparently controlled only by elements in the 5′-region of the gene (**Table 1**) (Lai et al., 2002).

Non-C_4_-associated NADP-ME isoforms targeted to the chloroplast in C_3_ and C_4_ plants also appear to be involved in defense responses, as well as providing pyruvate and NADPH for lipid and amino acid biosynthesis, and nitrogen assimilation (**Table 1**) (Maurino et al., 2001; Drincovich et al., 2011; Maier et al., 2011; Saigo et al., 2013). Phylogenetic and genomic analyses have shown that in independent C_4_ grass lineages, the same ancestral ortholog was used as the template for evolution of the C_4_-associated NADP-ME isoform, and that positive selection acted on particular codons, which resulted in adaptive parallel changes in the cognate proteins (Christin et al., 2009). These observations support the idea that the number of paths leading to the evolution of a C_4_-associated enzyme is constrained by the subcellular compartment and milieu in which it must function, and that, more generally, if suitable evolutionary enablers were not present in the C_3_ ancestor, then the C_4_ syndrome could not evolve (Christin and Osborne, 2013; Christin et al., 2013).

### NAD-Malic Enzyme

Although malate is the substrate of NAD-ME, in C_4_ species using this enzyme as the primary decarboxylase, Asp is actually the first stable organic acid formed. The C_4_-associated NAD-ME is a heterodimer made up of α and β subunits, and as described above, is highly active in the mitochondria of BS cells. However, NAD-ME isoforms are found in all plant mitochondria, where they play a key role in the respiration of malate in the tricarboxylic acid (TCA) cycle (**Table 1**) (Drincovich et al., 2011; Maier et al., 2011). Given the ubiquitous nature of this decarboxylase, it is interesting that not more is known of the evolutionary history of the C_4_-associated enzyme in any C_4_ species. The number of active NAD-ME isoforms is also not clear for many C_3_ and C_4_ species that have been examined.

Regulatory sequences directing BS-specific expression have been identified in the 5′-end of the coding regions of the NAD-ME α and β subunit genes from the C_4_ species *Gynandropsis gynandra* (previously *Cleome gynandra*; **Table 1**) (Brown et al., 2011). As corresponding sequences from the orthologous genes of *Arabidopsis thaliana* also drive expression in the BS of *G. gynandra*, it appears that the specific NAD-ME expression pattern of C_4_ species likely resulted from changes affecting the activity of a *trans*-acting factor (Brown et al., 2011).

### PEP Carboxykinase

ATP-dependent PCK isoforms are found in all plants. They are cytosolic enzymes and in C_3_ species, have been found to play significant roles in numerous metabolic processes that require or utilize PEP or OAA (**Table 1**). These include the mobilization of carbon from lipids and amino acids in seeds during gluconeogenesis and seedling development (Leegood and ap Rees, 1978), the control of stomatal aperture (Penfield et al., 2012), replenishment of TCA intermediates (Walker et al., 1999), pH regulation (Walker et al., 1999, 2001; Chen et al., 2004), synthesis of amino acids, and metabolism of nitrogenous assimilates during transport to storage tissues (Walker et al., 1999; Delgado-Alvarado et al., 2007).

In some PCK-type species, gene families have been identified (Finnegan et al., 1999; Christin et al., 2008) although overall, there is limited information regarding the size of the PCK gene family in both C_3_ and C_4_ plants, the evolutionary origin of the C_4_-associated PCK isoforms, and the regulatory mechanism(s) responsible for BS expression. Some of this lack of knowledge can be explained by the few species that have been identified as utilizing PCK as their primary decarboxylase, and the lack of information on the closest C_3_ relative of the C_4_ species that have been classified as PCK-subtype. A phylogenetic and genomic study has indicated that in the monocots, gene duplication events gave rise to different PCK gene lineages, and as for NADP-ME, convergence is seen, with parallel adaptive amino acid changes in the C_4_ isoforms in distinct C_4_ lineages, suggesting a predisposition of these orthologs for “C_4_-ness” (Christin et al., 2009).

## Flexibility in Organic Acid Production and Utilization in C_4_ Plants

The observation that in each C_4_ species, the activity of one 4-C organic acid decarboxylating enzyme predominates in leaf tissue offered a simple criterion by which to categorize C_4_ plants, and stimulated comparative analyses between and within C_4_ subtypes at the leaf anatomical, biochemical, physiological, and molecular biological levels. For example, correlations have been made between leaf anatomy and primary C_4_ decarboxylases (Gutierrez et al., 1974; Hatch et al., 1975; Kanai and Edwards, 1999; Edwards and Voznesenskaya, 2011). However, work with a number of C_4_ species has also shown that in addition to the primary decarboxylase, there is increased activity of another of the C_4_ decarboxylases (Gutierrez et al., 1974; Meister et al., 1996; Kanai and Edwards, 1999; Wingler et al., 1999; Muhaidat et al., 2007; Pick et al., 2011; Sommer et al., 2012; Muhaidat and McKown, 2013; Bräutigam et al., 2014; Koteyeva et al., 2015), and increased levels of the 4-C organic acid substrate (Kortschak et al., 1965; Hatch et al., 1967; Hatch, 1971; Meister et al., 1996; Pick et al., 2011; Sommer et al., 2012). This was recognized early on for C_4_ species that use Asp as the transferred organic acid and PCK as the primary decarboxylase, where malate levels and NAD-ME activity are also appreciable (Edwards et al., 1971). However, even in species such as sugarcane and maize, clear NADP-ME subtypes based on the above criteria, significant Asp formation and PCK activity were detected in the early ^14^CO_2_ labeling studies (Kortschak et al., 1965; Hatch and Slack, 1966). Both empirical and modeling studies now argue that nearly all C_4_ plants transfer more than one 4-C and 3-C organic acid during CO_2_ assimilation, and alternative decarboxylation pathways function in most C_4_ species (Pick et al., 2011; Sommer et al., 2012; Bellasio and Griffiths, 2014; Kromdijk et al., 2014; Wang et al., 2014). Insights into the consequences of C flux through the alternative pathways on plant metabolism suggest they extend beyond the obvious provision of CO_2_ for carbohydrate production, and likely impact on the ability of C_4_ plants to cope with diverse and fluctuating environments (Furbank, 2011; Bellasio and Griffiths, 2014; Kromdijk et al., 2014; Sharwood et al., 2014; Stitt and Zhu, 2014; Wang et al., 2014), and potentially play a role in development (Sommer et al., 2012).

## Maize – A Case Study in Flexibility of C_4_ Acid Production and Utilization

**FIGURE 5:**
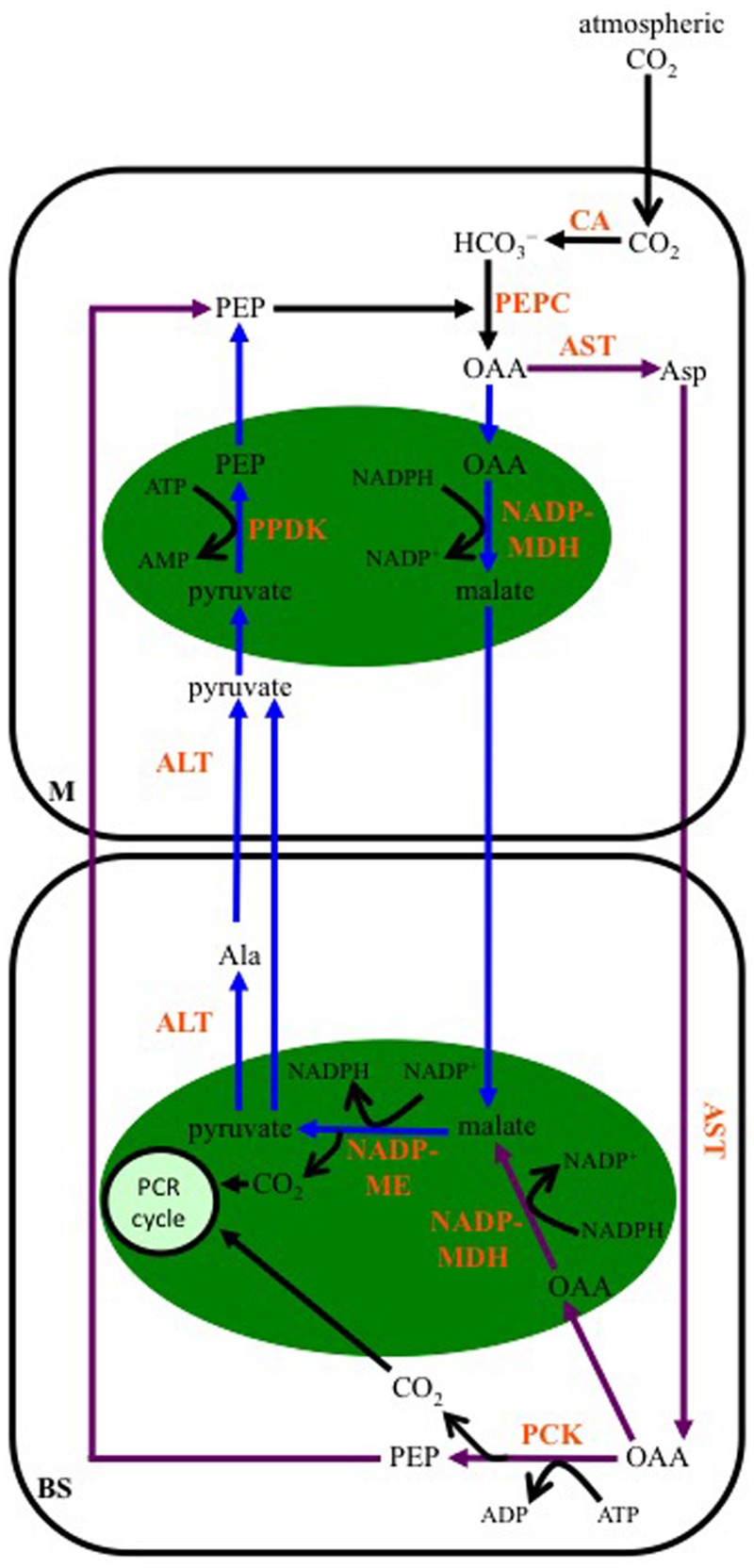
**Schematic of the proposed carbon flow in maize leaves when both malate and Asp are the transferred 4-carbon organic acids.** The initial steps of carbon flow are identical, with CA activity converting atmospheric CO_2_ to bicarbonate (HCO_3_^-^), and PEP being carboxylated by PEPC to produce OAA in M cells. Purple arrows: When Asp is transferred to BS cells, there is no net movement of reducing equivalents to the BS. ATP is required for PCK activity in the BS cytosol; however, there is reduced ATP demand in the M for PEP regeneration. This is the scenario predicted when the absorbed irradiance is relatively high in BS cells (Kromdijk et al., 2014; Wang et al., 2014). Blue arrows: When light striking a leaf is preferentially absorbed by the M, the flux through PCK decreases. Malate and reducing equivalents are transferred to the BS. Demand for ATP is lowered in BS cells due to reduced PCK activity. However, ATP requirements increase in M cells for PEP regeneration from pyruvate. ALT, Ala aminotransferase; AST, Asp aminotransferase; NADP-MDH, NADP-malate dehydrogenase; NADP-ME, NADP-malic enzyme; PCR cycle, photosynthetic carbon reduction cycle; green ovals, chloroplasts. Pathways modified from Furbank (2011), Pick et al. (2011) and Wang et al. (2014).

Early studies using ^14^CO_2_ to label actively photosynthesizing maize leaves showed that about 75% the label was quickly incorporated into malate, and 25% into Asp at a slower rate (Kortschak et al., 1965; Hatch and Slack, 1966; Hatch et al., 1967; Hatch, 1971). Significant PCK activity was subsequently found in maize leaves (Walker et al., 1997) and isolated BS cells (Wingler et al., 1999). The scheme of decarboxylase activities in maize leaves proposed by Wingler et al. (1999) included the “classical” NADP-ME pathway with the decarboxylation of malate in BS chloroplasts by NADP-ME, but also the transamination of Asp to OAA in BS mitochondria, followed by the release of CO_2_ in the BS cytosol by PCK, using ATP, the origin of which was unresolved. Later proposals have suggested that both malate and Asp are produced from OAA either in the M chloroplasts via NADP-MDH or through cytosolic AST activity (Pick et al., 2011), and OAA resulting from Asp transamination in the BS may be either decarboxylated directly by PCK in the BS cytosol, using ATP generated from chloroplast activities (Furbank, 2011), or transported into BS chloroplasts, reduced to malate by NADP-MDH, with the malate then decarboxylated by chloroplastic NADP-ME (**Figure 5**) (Furbank, 2011; Pick et al., 2011; Wang et al., 2014).

The transfer of malate and Asp result in different energy scenarios in the M and BS cells of maize. NADP-ME-type species, like maize, are typically described as having fewer grana in BS chloroplasts than in chloroplasts of M cells (Gutierrez et al., 1974; Hatch et al., 1975; Kanai and Edwards, 1999; Edwards and Voznesenskaya, 2011), indicating limited capacity for PSII activity and NADPH production via linear electron transport in the BS (Leegood et al., 1983; Hatch, 1987; Meierhoff and Westhoff, 1993). As a result, about half the 3-PGA produced by Rubisco in BS chloroplasts is transported to the M for phosphorylation and reduction to triose phosphate (triose-P; Hatch, 1987). Some of the triose-P is then returned to the BS for regeneration of ribulose-1,5-bisphosphate and starch production, while the rest is used in carbohydrate synthesis in M chloroplasts. When malate is decarboxylated by NADP-ME, reducing equivalents are moved from the M to the BS, contributing to the NADPH requirements of triose-P generation in the BS (**Figure 5**). The pyruvate returned to the M following NADP-ME decarboxylation is used by PPDK to generate PEP in a reaction that uses ATP. This adds to the ATP demand in the M over that needed for triose-P production. In the case of Asp being transferred to the BS, no reducing equivalents are moved, and ATP is required by PCK in the BS for the decarboxylation of OAA; however, the need for ATP is lessened in the M as PEP is returned to these cells following PCK activity (**Figure 5**).

Clearly, for efficient functioning of the maize C_4_ pathway overall, coordination of ATP and reducing equivalent supply and use must occur between the M and BS. In this regard, it has been proposed that the ability to move carbon through both malate and Asp decarboxylation pathways plays a role in adjusting M and BS energy balance to facilitate efficient functioning of maize C_4_ photosynthesis in diverse and changing environments, and during development (Furbank, 2011; Pick et al., 2011; Bellasio and Griffiths, 2014; Kromdijk et al., 2014; Stitt and Zhu, 2014; Wang et al., 2014). It has also been postulated that the large pools of 3-C and 4-C organic acids, which support their diffusion between M and BS cells in C_4_ species (Leegood, 1985; Stitt and Heldt, 1985), act as reserves of ATP and NADPH to buffer against rapid changes in light availability over longer time scales than is possible for C_3_ plants (Stitt and Zhu, 2014). Being able to switch between the species of 4-C and 3-C organic acids that are transferred, with their differing contributions to M and BS energy balance, strengthens this buffering capacity, and the ability to maintain efficient CO_2_ assimilation. The sharing of C flux between multiple metabolites is also seen as a benefit as it allows the concentration and diffusion gradient of individual 3-C and 4-C organic acids to be lower without compromising overall C_4_ cycle activity (Pick et al., 2011; Kromdijk et al., 2014; Wang et al., 2014).

So are the suggested advantages of operating a combination system of transferred organic acids and decarboxylating enzymes in maize realized? Experimental and modeling studies have begun to address this question with respect to differing light environments. Sharwood et al. (2014) looked at the effects of shading on maize NADP-ME and PCK activities along with other photosynthetic characteristics. As expected, relative to control plants, overall biomass and photosynthetic rates of the shade plants were significantly lower, and in addition, both NADP-ME and PCK amount and activities were reduced in the shade-grown individuals. Interestingly however, PCK activity was decreased to a greater extent, with a 75% reduction versus a 60% drop for NADP-ME (Sharwood et al., 2014). Although energy partitioning between M and BS cells, BS PSII activity, and/or grana stacking in the chloroplasts of the two cell types were not investigated, the results do indicate that light availability differentially affects the 4-C organic acid produced, and C flux through the two decarboxylation pathways utilized by maize leaves, and consequently the balance of energy in M and BS cells.

Two recent modeling studies examined the effects of differing light regimes on M and BS energy partitioning while considering combination transfer organic acids and decarboxylating pathways (**Figure 5**) (Bellasio and Griffiths, 2014; Wang et al., 2014). A novel metabolic model along with experimentally measured inputs (Bellasio and Griffiths, 2014) showed that ATP and NADPH production were complementary in maize M and BS cells such that changes in light quality leading to increases in ATP or reducing power production in one cell type were offset by decreases in the other. This work supported the idea that the presence of alternative decarboxylation pathways, with their differing contributions to M and BS cell energy demands, are important in maintaining cell-type energy balance and high leaf CO_2_ assimilation rates in different light conditions (**Figure 5**) (Bellasio and Griffiths, 2014). A systems modeling approach taken by Wang et al. (2014) looked at CO_2_ assimilation rate for combination systems of transfer acids and decarboxylases relative to the standard NADP-ME pathway when differing amounts of light were allocated to the M and BS. This analysis also showed that flexibility in the organic acids transferred between M and BS cells, and the associated decarboxylase activities, as now recognized as operational in maize, would ensure high photosynthetic efficiency in differing light environments.

The proposal that a combination of transfer acids and decarboxylase pathways might contribute to the ability of C_4_ leaf organic acid pools to act as capacitors of ATP and reducing power equivalents in fluctuating light environments (Stitt and Zhu, 2014) has not been directly tested. However, the systems modeling approach described above (Wang et al., 2014) did support the suggestion that the concurrent transfer of multiple organic acids reduced the concentration gradient of individual species needed for C_4_ pathway function. The concentration gradients between M and BS cells for the standard NADP-ME pathway, and the combination pathway now recognized in maize leaves were simulated. A slight decrease in the malate gradient was predicted for the combination pathway compared to the standard NADP-ME pathway, while the pyruvate gradient was reduced by about 20%. Concentration gradients for Asp, PEP and Ala were predicted to compensate for these reductions and ensure efficient C_4_ photosynthesis (Wang et al., 2014).

## Conclusion and Perspectives

The identification of dicarboxylic acids as the initial stable products of photosynthesis in some plant species was the first recognition of photosynthetic diversity in the terrestrial plant world. This discovery opened up not only the field of C_4_ biochemistry, but also all aspects of C_4_ plant biology, including a multitude of comparative studies in the areas of anatomy, physiology, ecology, evolution, biogeography, and recently, omics. Much of present day C_4_ photosynthesis research is focused on understanding the steps in the evolution of the pathway with an aim of transferring it into C_3_ crop plants to increase yield and/or mitigate effects of climate change.

Four- and 3-C organic acids are the substrates and products of the C_4_ acid transfer cycle that links CO_2_ uptake with CO_2_ fixation into carbohydrates. In most C_4_ plants, these reactions take place over two cell types and function to concentrate CO_2_ in internal cells of a C_4_ leaf. The genes encoding the decarboxylases that catalyze the release of CO_2_ from the 4-C organic acids near the sites of fixation and carbohydrate production have been co-opted for the C_4_ pathway from ancestral C_3_ genes. Historically, three subtypes of C_4_ plants have been recognized, based on the transferred 4-C acid and the decarboxylase with the highest activity in leaf tissue; however, recent work suggests that the majority of C_4_ plants transfer more than one type of 4-C acid, as well as multiple 3-C acids, during CO_2_ fixation, and have significant activity of the required secondary decarboxylase. Modeling studies indicate that the evolutionary routes to the C_4_ syndrome favored combination pathways.

The realization that multiple organic acids are transferred and combination decarboxylation pathways exist in C_4_ species has expanded our conception of C_4_ plant metabolism. However, we have only begun to comprehend the consequences of this more complex biochemistry on the overall biology of a C_4_ plant. Increasing evidence indicates that combination pathways allow flexibility in differing light regimes to meet the energy demands of the M and BS cells for CO_2_ fixation, thereby ensuring efficient functioning of the C_4_ CO_2_ concentrating mechanism. However, little to no information is available on how combination pathways may enable C_4_ plants to mitigate the effects of other fluctuating environmental factors or stresses, or how they may play a role during development.

Future work with C_4_ plants should consider the effects of differing light environments, nutrient availabilities, salinity, and leaf development on the levels of organic acids and other metabolites, enzyme and photosystem activities, CO_2_ assimilation rates, leaf anatomy, chloroplast ultrastructure, and M and BS energy status. The inter- and intracellular location of AST and ALT isoforms, and the identification of additional transporters on organelle membranes would contribute to the clarification of actual paths of C flux. Future studies should also consider the evolutionary history of C_4_-associated NAD-ME and PCK isoforms and identify the molecular changes responsible for their expression, location and activity. With C_4_ species, along with groups containing closely related C_3_, C_3_–C_4_ intermediate, and C_4_-like species, increasingly being examined in genomic, transcriptomic, proteomic, metabolomic, and flux studies, the resolution of the components and mechanisms of combination pathways will be a focus for future research. All of the above multifaceted approaches will allow a more comprehensive understanding of the costs and/or benefits of operating combination pathways on C_4_ plant metabolism, growth and productivity. In turn, the knowledge will give insights into the significance of these systems on C_4_ plant physiology and ecology, and contribute to attempts to increase C_4_ crop yield and ensure global food security, predict the effects of different climate change scenarios on natural and agricultural C_4_ species-rich environments, and inform future strategies in plant biotechnology.

## Author Contributions

ML wrote the entire review and contributed all of the intellectual content.

## Conflict of Interest Statement

The author declares that the research was conducted in the absence of any commercial or financial relationships that could be construed as a potential conflict of interest.

The reviewer GG, and handling Editor declared their shared affiliation, and the handling Editor states that the process nevertheless met the standards of a fair and objective review.
